# Supplementary material for: Evaluation of biochemical, physiological traits and percentage of essential oil of sickleweed (Falcaria vulgaris) population in different geographical and climatic regions
Source: PLoS One. 2023 Jun 21;18(6):e0287480. doi: 10.1371/journal.pone.0287480 (PMC10284402; doi:10.1371/journal.pone.0287480)
Supplement: Attachment — Submitted filename: reponse to Edits Amendment Required.pdf [file pone.0287480.s001.pdf]

Dear Editor

Thank you for your useful comments and suggestions. Also, detailed corrections are listed below point by point.

1. 1. We suggest you thoroughly copyedit your manuscript for language usage, spelling, and grammar. If you do not know anyone who can help you do this, you may wish to consider employing a professional scientific editing service.

The article was edited in terms of writing and grammar. Dr. Mojtaba Kordrostami edited the article and has expertise in this work.

A copy of your manuscript using track changes uploaded as a *supporting information* file and also A clean copy of the edited manuscript uploaded as the new *manuscript* file.

Thanks once again.

Best Regards

On behalf of the author
