# Supplementary material for: Evaluation of biochemical, physiological traits and percentage of essential oil of sickleweed (Falcaria vulgaris) population in different geographical and climatic regions
Source: PLoS One. 2023 Jun 21;18(6):e0287480. doi: 10.1371/journal.pone.0287480 (PMC10284402; doi:10.1371/journal.pone.0287480)
Supplement: Attachment — Submitted filename: Response to Reviewers.docx [file pone.0287480.s002.docx]

**Dear Editor**

Thank you for your useful comments and suggestions on the language and structure of our manuscript. We have modified the manuscript accordingly and showed in the revised MS by yellow highlighted and Track Changes. Also, detailed corrections are listed below point by point.

Thanks once again.

Best Regards

On behalf of the authors

**ACADEMIC EDITOR:**

Abstract of the manuscript must be revised highlighting the methodology and results clearly. This section should contain all key results, and conclusion which is missing.

Introduction needs some careful attention. All the abbreviations on first appearance must be presented in full. Language is generally poor. In some places is it not quite clear what the authors meant to say. Line 13 perennial and perennial??? Line 14 genus Falcaria. Genus should be italicized. Moreover, population Gilan-Deylaman and Kur- 29 Gerger-e Sofla were also identified as superior populations??? Not clear. Units are missing in different parts of manuscript like in table 1 Height above sea level. Author mentioned in line 64 the importance of Identification of morphological diversity but not describe in the manuscript. Climatic factors are not investigated as a main objective of your work.   
Material and methods need improvement. This section is weak, we need complete procedures. All phytochemical analyzed in the study should be explained separately with subheadings like: Sample collection technique (plant collection): Sample Preparation: as each assay has a specific method of sample preparation or extraction. Phytochemicals: Each assay should be explained in this section with subheadings such as, Total chlorophyll: Total carotenoids:

Results are not discussed in appropriately. Discussion needs improvement with more references to support your findings.

Conclusions are not in support of your findings. Significant findings should be mentioned with recommendation.

Answer: All the things requested by the respected editor were done in the text and the article was corrected accordingly.

**Reviewer comments**

**Reviewer #1**

The authors conducted a study entitled "Evaluation of biochemical, physiological traits and percentage of essential oil of sickleweed (Falcaria vulgaris) population in different geographical and climatic regions"

I regret to inform you that I cannot recommend this work for publication in this prestigious journal.

For me the study is limited and important information should be inserted in the MS, the chemical composition of the essential oils should be investigated, a practical application of these oils and the correlation with the different collection times should be investigated, the information brought in the work is not sufficient to justify its publication.

**Answer**: In this article, the aim was not to investigate the chemical composition of the essential oil, and of course the opinion of the respected referee is correct and it can be a new article. The main goal was to investigate the effect of environments with different altitude, temperature and climate on biochemical traits and percentage of essential oil.

Graduate University of Advanced Technology (Kerman, Iran) to support this project.

**Reviewer #2**

Reviewer #2: Although samples from different environments cannot fully reflect the true genetic information, it also can screen out the most suitable populations of the same species in a given place. There are still some minor problems in the manuscript that need further revision

1.In the introduction, the goal of breeding is not stated clearly. In line 84, what aspects of the best populations with breeding potential are considered and what are the criteria? It should be stated clearly in the preceding introduction.

**Answer**: Corrected and revised in text

2. in L151, what is the full name of PCV? phenotypic coefficient variation？Abbreviations should be given where the manuscript first appears

**Answer**: Corrected in text

3.in L213-226, the specific traits of the three main groups are not described separately and are not marked in Figure 1.

**Answer**: Corrected in text

4.The results in this manuscript are not well compared and analyzed with those already reported.

**Answer**: Corrected in text

5.There is no detailed discussion on how the suitable or superior population is judged and compared, and what are their distinguishing features from other populations.

**Answer**: revised in text

We would like to give you a special thanks for your careful reading of the whole MS and also for your meaningful corrections that helped us a lot to improve our manuscript significantly. We have revised the manuscript according to your suggestions and the other reviewer’s suggestions and highlighted.

Best Regards

On behalf of the authors
