# Supplementary material for: Evaluation of biochemical, physiological traits and percentage of essential oil of sickleweed (Falcaria vulgaris) population in different geographical and climatic regions
Source: PLoS One. 2023 Jun 21;18(6):e0287480. doi: 10.1371/journal.pone.0287480 (PMC10284402; doi:10.1371/journal.pone.0287480)
Supplement: Attachment — Submitted filename: Manuscript_Reviewer.docx [file pone.0287480.s003.docx]

**Evaluation of biochemical, physiological traits and percentage of essential oil of sickleweed (*Falcaria vulgaris*) population in different geographical and climatic regions**

**Mehdi Rahimi^1*^, Mojtaba Kordrostami^2*^ and Jaber Nasiri^2^**

^1^Department of Biotechnology, Institute of Science and High Technology and Environmental Sciences, Graduate University of Advanced Technology, Kerman, Iran. ORCID: 0000-0001-5625-3275

^2^Nuclear Science and Technology Research Institute (NSTRI), Nuclear Agriculture Research School, P.O. Box 31485498, Karaj, Iran.

*Correspondence should be addressed to Mehdi Rahimi, [me.rahimi@kgut.ac.ir](mailto:me.rahimi@kgut.ac.ir), [mehdi83ra@yahoo.com](mailto:mehdi83ra@yahoo.com); Mojtaba Kordrostami, mkordrostami@aeoi.org.ir

**Abstract**

Sickleweed (*Falcaria vulgaris*) is the name of a species of annual, and perennial herbaceous plants of the genus *Falcaria*. Climate change could negatively influnces the performance of various plant species in plant kingdom. In this study, 15 different sickleweed populations from seven provinces of the country were collected based on an unbalanced nest design with 10 replications and the percentage of essential oil, types of chlorophyll, phenol, proline, protein, and carotenoids were measured on them. Results ~~of the analysis of variance~~ showed that there was a significant difference between populations at the level of one percent for the studied traits. The results of the mean comparison showed that populations Ard-Shaban and Qaz-Ilan in terms of the evaluated traits and especially the percentage of essential oil were at the upper of the studied samples and selected as suitable populations. In addition, populations Gilan-Deylaman and Kur-Gerger-e Sofla were also identified as superior populations in terms of studied traits by cluster analysis and principle component analysis (PCA). Since the high level of proline and biochemical and physiological traits in plants can play a role in plants' tolerance to stresses, therefore, populations with high values of these traits can be used in stress tolerance breeding programs. Therefore, in this study, populations Gilan-Deylaman and Kur-Gerger-e Sofla can be suitable populations for this purpose. In addition, the essential oil of this plant is used in the treatment of diseases, and therefore populations Ard-Shaban and Qaz-Ilan, which showed a high percentage of essential oil, can be used in breeding programs to increase the performance of this trait.

Keywords: Cluster analysis; Diversity; *Falcaria vulgaris*; Principal component analysis.

**Introduction**

Today, the nutritional, health, and economic value of medicinal plants, along with the role of 25% in the production of drugs in the world, has led to considerable attention to them [1]. The country of Iran has a variety of plant species due to its climatic diversity. The genetic resources available in each country are one of the most valuable potential agricultural resources of that country. The main goal of studying the diversity between and within different plant populations and the relationship between germplasm collections is to finally use this information for the production and development of cultivars with better productivity than cultivated species [2], [Add ref *]. In general, genetics affects plant yield and secondary metabolites of medicinal plants, and the interaction of area×mass is very significant on the yield of total dry matter and plant and varies depending on the region and type of mass [3, [2], Add ref **]. Transferring masses and different species from one region to another as well as the forcing of the adaptation plant to the new environment can change the ratio of secondary metabolites and in some cases induce new medicinal metabolites [4].

ref *

<https://doi.org/10.1016/j.rhisph.2023.100706>

<https://doi.org/10.1007/s42729-022-00916-7>

<https://doi.org/10.1080/03650340.2019.1619078>

ref **

<https://doi.org/10.1007/s42729-021-00485-1>

<https://doi.org/10.1080/00103624.2018.1510949>

Climate, directly and indirectly, affects all factors affecting the environment and plant growth [Add ref ***]. Experiments conducted in recent years show the effective role of climatic factors on the distribution of plants [5]. According to some studies, although the weather factor plays the most important role in the growth and distribution of plants, plants are developed through several different factors including climate, soil characteristics, and natural conditions [6]. Sickleweed (*Falcaria vulgaris*) belongs to the *Falcaria* genus and the Apiaceae family and belongs to the Eudicots Clade. Sickleweed is especially important because of its nutritional value and medicinal effects on the human body. This plant is usually consumed as a vegetable and is a common food in spring. It is recommended in traditional medicine books to treat skin wounds, stomach problems, liver diseases, and blood purification, and its therapeutic effects have been proven in modern medical science [7]. The increasing recognition of the properties of this plant species and the increasing population growth along with the decrease in rainfall have put increasing pressure on this plant population that preventive actions should be done for the protection and breeding of this plant.

ref ***

<https://doi.org/10.1007/s10343-021-00590-2>

<https://doi.org/10.1007/s10343-020-00535-1>

<https://dx.doi.org/10.21608/ajs.2018.34147>

There are different methods to investigate the diversity between different plant samples; one of the cheapest and most common methods is the morphological diversity investigation. Although morphological diversity is influenced by environmental conditions compared to molecular diversity, it is widely used in different plants. Identification of morphological diversity is not only useful in the management of plant germplasms but also provides a good idea to researchers for plant breeding [8]. Moreover, one of the important goals of medicinal plants is to select populations with high levels of secondary metabolites and increase the number of important compounds in these plants, which is possible by identifying effective physiological and biochemical parameters and knowing the relationships between plants and environmental factors [9]. Geographical and climatic factors are effective in the production of secondary metabolites and morphological characteristics of medicinal plants. Although effective substances are made by guiding genetic processes, their production is significantly influenced by environmental factors such as light, temperature, and altitude [].. Environmental factors affect the total amount of effective substances, their constituent elements, dry weight production, and plant morphology [10].

Various studies have been conducted in the field of investigating biochemical and physiological traits under the influence of various factors such as different regions, different environmental conditions, etc., in different medicinal plants [11-14]. In the case of the medicinal species Satureja, Spear Thistle, Persian hogweed, and chicory, it was shown that there is a direct relationship between the increase in height and the amount of phenolic and flavonoid active substances [15].

Research have revealed that there might be significant regional variation in the biochemical characteristics of different plants. For instance, it has been discovered that arid regions with little rainfall and high temperatures have higher levels of medicinal plants in terms of their total phenolic and flavonoid content, antioxidant activity, and essential oil content. The influence of environmental elements like temperature, rainfall, and soil type can be used to explain the diversity in the biochemical characteristics of medicinal plants across various geographical areas [16-20].

So far, not much study has been done in the world and Iran regarding the evaluation of morphological traits of different populations of sickleweed plants and the determination of their morphological identification. The biochemical and physiological traits of sickleweed vary across different populations, depending on the environmental factors such as temperature, rainfall, soil type, and altitude. Therefore, this study aims to evaluate the effect of geographical origin on populations and to investigate the diversity between populations, as well as to identify the best populations with breeding potential with the highest yield in terms of important traits such as essential oil, and physiological and biochemical traits. In addition, the use of this information is for creating a future protection program to prevent them from becoming extinct.

**Materials and Methods**

**Climatic factors**

The climatic characteristics of the regions are shown in Table 1. The information related to the height of the location as well as the geographic longitude and latitude was recorded by Google Earth software, and the information related to the weather as well as various bioclimatic parameters [21] was estimated from the WorldClim website.

**Plant collection**

The 15 different plant samples (Table 1) were collected from seven provinces of Iran. These seven provinces include Ardabil, Kurdistan, Kermanshah, Gilan, Hamedan, Qazvin, and Qom so the number of selected regions was different for each province. For each population in each region, 10 different samples were selected and considered as replicates. In addition, the evaluation of traits and measurements was performed on these samples, and studies were conducted based on a nesting design with an unbalanced number of regions. In each area, the distance of the samples from each other was at least 10 meters to avoid interference from the samples.

| **Table 1. Characteristics of the collection areas of Sickleweed plant samples** | | | | | | |
| --- | --- | --- | --- | --- | --- | --- |
| Row | Province | City | Region | Longitude (degree) | Latitude (degree) | Height above sea level (meter) |
| G1 | Kermanshah | Sonqor | Kivananat | 47.27889 | 34.84972 | 1847 |
| G2 | Kermanshah | Sonqor | Bavaleh | 47.69778 | 35.02722 | 2010 |
| G3 | Kermanshah | Sahneh | Sahneh | 47.75833 | 34.44194 | 1465 |
| G4 | Hamedan | Asad abad | Chaharduli | 48.065 | 34.93139 | 1890 |
| G5 | Kurdistan | Gorveh | Panjeh Ali | 47.70834 | 35.18667 | 1912 |
| G6 | Kurdistan | Dehgolan | Bolbanabad | 47.41195 | 35.15222 | 1851 |
| G7 | Kurdistan | Dehgolan | Amirabad | 47.31084 | 35.10361 | 1974 |
| G8 | Kurdistan | Kamyaran | Gerger-e Sofla | 47.27667 | 35.00334 | 1824 |
| G9 | Kurdistan | Kamyaran | Qaleh Gah | 47.21196 | 34.95001 | 1611 |
| G10 | Kurdistan | Bijar | Nemat abad Auliya | 47.41333 | 35.68444 | 1922 |
| G11 | Kurdistan | Bijar | Seylatan | 47.83054 | 36.03666 | 1617 |
| G12 | Qazvin | Qazvin | Ilan | 50.64778 | 36.42528 | 1450 |
| G13 | Ardabil | Meshkinshahr | Shaban | 47.44833 | 38.37556 | 1236 |
| G14 | Qom | Qom | Khalajastan | 50.19028 | 34.67944 | 1952 |
| G15 | Gilan | Siahkal | Deylaman | 49.90528 | 36.88889 | 1455 |

**Sample preparation**

The leaves of the plants were collected in the vegetative stage of the Sickleweed plant in the early and mid-spring of 2021, and the fresh leaves were placed in foil after harvesting, placed in liquid nitrogen, and transferred to the laboratory for use in the next experiments such as percentage of essential oil, measuring physiological and biochemical traits based on methods described by Sudhakar et. al, [22].

**Chlorophyll content**

Total chlorophyll, as well as chlorophylls a and b, were measured by Arnon's method [23] using 0.2 g of fresh plant leaf material in a test tube and grind with 10 mL of 80% acetone, and the absorbances read at 663 and 645 nm in a spectrophotometer. Calculate the amount of chlorophyll present in the extract in mg chlorophyll per g tissue using the following equations:

(1)

$${mg chlorophyll a}/{g tissue}=[\left( 12.7\times A663 \right)-\left( 2.69\times A645 \right)]\times\frac{v}{1000\times w}$$

(2)

$${mg chlorophyll b}/{g tissue}=[\left( 22.9\times A645 \right)-\left( 4.68\times A663 \right)]\times\frac{v}{1000\times w}$$

(3)

$${mg total chlorophyll}/{g tissue}=\left[ \left( 20.2\times A645 \right)-\left( 8.02\times A663 \right) \right]\times\frac{v}{1000\times w}$$

Where:

A = absorbance at specific wavelengths

V = final volume of chlorophyll extract

W = fresh weight of tissue extracted

**Total carotenoids**

The total carotenoids were measured by Price and Hendry's method [24] using the sample extract, obtained for the estimation of chlorophylls by the Acetone Method. The absorbances are read at 663 nm, 645 nm, and also at 480 nm in a spectrophotometer and the concentration of pigments is mg/g fresh weight. Carotenoid content is calculated using the following equations.

$${mg total carotenoids}/{g tissue}=[A480+\left( 0.114\times A663 \right)-\left( 0.638-A645 \right)]\times\frac{v}{1000}\times w$$

(4)

This equation compensates for the interference at this wavelength, due to chlorophyll.

**Proline**

The Bates et al. method [25] was used to measure the proline content of leaves. Initially, 2 ml of the supernatant after centrifuging the extract was combined with 2 ml of the reagent ninhydrin and 2 ml of pure acetic acid, then the mixture was placed in an air bath at 100°C for 1 hour. The tubes containing the mixture were then as soon as possible put in the ice bath. The mixture was then given 4 ml of toluene, and the tubes were thoroughly vortexed. The tubes were stacked for 15 to 20 minutes to create two distinct layers. Proline concentration was determined using the toluene and proline-containing upper color phase. The absorbance was determined at 518 nm and the amount of proline was calculated using a standard curve.

**Protein**

The proteins were extracted from the aerial parts at a temperature between 0 and 4 degrees Celsius in ice water with phosphate buffer. Then, the Coomassie Brilliant Blue (CBB) G-250 was used to give a consistent blue color. After 25 min, the absorbance was read by a spectrophotometer at 595 nm. Protein concentrations were calculated using a standard curve to measure the amount of total protein by Bradford's method [26].

**Reducing sugars**

In a Chinese mortar, 0.02 g of the plant's aerial section was ground with 10 ml of distilled water and heated on an electric stove. Once the solution had boiled, the heat was turned off, and filter paper was used to collect the solution. Test tubes were filled with two milliliters of the produced extract, and two milliliters of copper sulfate solution, and then the test tubes' ends were sealed with cotton before being submerged in a hot bath at a temperature of 100 degrees Celsius for 20 minutes. Two milliliters of the phosphomolybdic acid solution were added to the tubes once they had cooled, and after a short while, a blue tint emerged. The test tubes were then agitated ferociously with a vortex device to evenly spread the color throughout the test tube. Using a spectrophotometer and the standard curve, the absorbance intensity of the solutions at 600 nm wavelength was measured by the Somogyi method [27] to show the amount of reducing sugars.

**Total phenolic**

The extraction of phenolic compounds was done according to Malik and Singh [28] and the stages of preparation, extraction, etc. are shown in Sudhakar et. al, [22]. The total amount of phenolic compounds were measured by the Folin-Ciocalteu method at 650 nm spectrophotometrically and expressed as mg phenols/100 g material.

**Essential oil**

The percentage of essential oil was also determined by distillation with water using Clevenger- type apparatus by the European Pharmacopoeia [29], and after drying the water with the essential oil using dry sodium sulfate, the amount of essential oil was purified and then the percentage of essential oils was calculated relative to the dry weight.

**Statistical analysis**

Statistical analyzes including descriptive statistics, analysis of variance, and multivariate analysis were performed on the data. Descriptive statistics including mean, range, and standard deviation, etc. were performed on the average data of 15 populations, and the phenotypic coefficient variation (PCV) was calculated as follows for the studied traits.

(5)

$$\mathrm{CV}_{p}=\left( {\sqrt{\sigma_{p}^{2}}}/\bar{x} \right)\times100$$

Before analysis, the variance of the data has checked for the correctness of the assumptions of the variance analysis, such as normal distribution of experimental errors, independence of experimental errors, uniformity of variances within the treatment, and the absence of interaction between treatment and provinces. After establishing these assumptions, the variance analysis of the data was carried out based on the unbalanced nested design, and Tukey's test was used to compare the mean. In addition, the variance components were calculated based on the expected value of the mean square of the unbalanced nested design. The measured data for the essential oil percentage, protein, sugar content, and proline traits were very small, so the first data were multiplied by 100 and then the obtained data were used and the variance analysis was performed using SAS software version 9.4 [30].

Multivariate analyses were used to investigate the genetic structure and grouping of populations and to identify kinship relationships between them. Grouping of sickleweed populations by using traits as well as grouping of the climatic regions by using bioclimatic parameters were done based on cluster analysis. The cluster analysis was done based on different methods and different distance criteria. Then, the distance method and the criterion that had the highest cophenetic correlation coefficient were selected and the grouping was done based on it. Finally, the distribution of the populations was also done by principal component analysis and the biplot method. The cluster analysis method and principal component analysis were done with PAST software [31]. Determining the number of groups was done with the Factoextra [32] package and R software.

**Results and Discussion**

Nineteen bioclimatic indicators were measured and calculated for the collection locations in this study to investigate the geographic variety. The diversity and similarity of these areas were then assessed using various cluster analysis techniques and distance criteria. Fig 1 displays the outcomes of the cluster analysis for these areas. The findings demonstrated that these locations are divided into two groups using the Elbow technique and that they share similar weather patterns and bioclimatic data. In the first group were Seylatan and Qaleh Gah regions from Kurdistan province, Sahneh from Kermanshah, Deylaman from Gilan, Ilan from Qazvin, and Shaban from Ardabil, and in the second group were Kiyunanat and Bavaleh regions from Kermanshah, Panjeh Ali, Bolbanabad, Amirabad, Gerger-e Sofla and Nemat abad auliya from Kurdistan, Khalajastan from Qom and Chaharduli from Hamedan. The range of a species geographic distribution is a complex issue in population ecology that cannot be fully understood. Generally speaking, whether a species can survive in its native environment depends on how well an individual can meet biological needs such as dietary, physiological, and behavioral needs [33]. Weather patterns and terrain, or more specifically, geographic diversity, have an impact on the quantity and richness of resources. The classic hypothesis of allopatric speciation postulates that physical obstacles like distances, lowlands, and heights, among others, can separate populations and lead to various trends in the variety of those populations [34, 35]. The alterations that distinct populations have endured along distinct evolutionary pathways may have resulted in a sort of superiority in reproduction and adaption to various places [36]. Plants grown in arid regions with low rainfall and high temperatures may have higher percentages of essential oil due to the need to conserve water and prevent water loss through transpiration [16, 20, Add ref ****].

ref ****

<https://doi.org/10.1007/s10343-021-00557-3>

<https://doi.org/10.1007/s40333-020-0020-y>

**[Fig 1 near here]**

Because the purpose of studying genetic populations is to compare genotypes in terms of the amount of existing variation, This way, a range of data can be used for the initial comparison between the studied genotypes and get a general view of the amount of existing variation. Descriptive statistics including the minimum, maximum, and range of the studied traits are shown in Table 2. The coefficient of variation parameter is one of the most important and valuable indices for estimating diversity in populations, and since this criterion is not affected by the trait measurement unit or its range, it is more important than other diversity criteria such as the range. Therefore, it is possible to make reliable selections to improve traits that have a higher coefficient of variation. Examining the phenotypic coefficient of variation of the traits showed that phenol, Chl. b and essential oil had the highest PCV. Therefore, these traits can be used in the breeding programs and effective selections can be made among the studied sickleweed populations to improve and breed these traits.

| **Table 2. Descriptive statistics of biochemical and physiological traits in Sickleweed populations** | | | | | | |
| --- | --- | --- | --- | --- | --- | --- |
| Traits | Statistical parameters | | | | | |
|  | Min | Max | Range | Avereage | Stand. dev | PCV |
| Essential oil (%) | 0.076 | 0.089 | 0.013 | 0.081 | 0.004 | 5.362 |
| Protein (mg/GFW) | 0.00679 | 0.00685 | 0.00006 | 0.00682 | 0.00002 | 0.23020 |
| Proline (mg/GFW) | 0.00245 | 0.00288 | 0.00043 | 0.00273 | 0.00011 | 4.19188 |
| Sugars (mg/GFW) | 0.04699 | 0.05737 | 0.01038 | 0.05530 | 0.00267 | 4.82979 |
| Chl. a (mg/GFW) | 7.381 | 8.397 | 1.017 | 8.014 | 0.364 | 4.545 |
| Chl. b (mg/GFW) | 2.018 | 2.516 | 0.498 | 2.198 | 0.119 | 5.426 |
| Total Chl. (mg/GFW) | 9.597 | 10.893 | 1.296 | 10.235 | 0.397 | 3.884 |
| Carotenoids (mg/GFW) | 4.152 | 4.608 | 0.456 | 4.434 | 0.163 | 3.685 |
| Phenol (mg/100 GFW) | 52.031 | 88.149 | 36.118 | 55.315 | 9.100 | 16.451 |

In addition, the lowest PCV is related to protein traits, and the improvement of this trait will be less successful than other traits through selection in the studied population. According to the values of the PCV of the traits, it is observed that there is a favorable variation among the traits for most of them, and they can be used to improve the studied populations of sickleweed (Table 2). The lowest PCV related to protein traits (0.23), and the highest related to phenol (16.45) and other traits were in this range. The reason for the high diversity of traits can be the environmental conditions and the different genetic backgrounds of the populations. The variation in leaf length trait and petiole length trait was more than other traits and therefore these traits can be considered by breeders because these traits play a role in stress tolerance.

The results of variance analysis of the unbalanced nest design for physiological and biochemical traits as well as the percentage of essential oil are shown in Table 3. The effects of the province and the population within the province for the studied traits showed a significant difference at the level of 0.01 between sickleweed populations (Table 3). The significance of the effects of the province and the population within the province showed that there is a significant difference between the provinces and between 15 different populations, and it shows the diversity among these populations and provinces.

| **Table 3. Unbalanced nest variance analysis of physiological and biochemical traits in Sickleweed populations** | | | | | | | | | | |
| --- | --- | --- | --- | --- | --- | --- | --- | --- | --- | --- |
| The mean square of traits | | | | | | | | | Df. | S.O.V. |
| Protein (mg/gfw) | Proline (mg/gfw) | Sugars (mg/gfw) | Chl. a (mg/gfw) | Chl. b (mg/gfw) | Total Chl. (mg/gfw) | Carotenoids (mg/gfw) | Phenol (mg/100gfw) | Essential oil (%) |  |  |
| 0.033 | 5.66E-03** | 2.722** | 21.086** | 1.387** | 15.436** | 4.306** | 3962.076** | 64.820** | 6 | Province |
| 0.006 | 4.79E-03** | 0.548** | 4.295** | 0.428** | 10.766** | 3.038** | 690.901** | 11.623** | 8 | Populations (Province) |
| 2.3E-07 | 4.28E-05 | 0.003 | 0.003 | 0.002 | 0.009 | 0.002 | 0.873 | 0.367 | 135 | Error |
| 0.18 | 0.98 | 1 | 0.68 | 1.73 | 0.92 | 0.99 | 1.47 | 8.83 | CV of design (%) | |
| **: Significant at 1% probability level | | | | | | | | | | |

Table 4 shows the results of variance components for the studied traits and the results showed that the percentage of variance components for the studied traits was between 25.31 and 83.92% among the studied populations and between 8.51 and 73.36% among the provinces. Based on these results, considerable diversity is observed between populations and between provinces and these differences can be used to select the best population. In addition, for some traits, variation within the population was also observed, which sees from the results of the percentage of variance components for the source of error. Variation within the population for these traits was less than 8%. Therefore, the chance of selecting superior individuals within populations will be low due to low diversity.

| **Table 4. Estimation of the variance components of the sources of variation in the unbalanced nest design of the studied physiological and biochemical traits** | | | | | | | | | |
| --- | --- | --- | --- | --- | --- | --- | --- | --- | --- |
| Traits |  | Sources of variation | | | | | | | |
|  |  | Province | |  | Populations (Province) | |  | Error | |
|  |  | Variance Components | % |  | Variance Components | % |  | Variance Components | % |
| Chl. a (mg/gfw) |  | 0.933 | 68.354 |  | 0.429 | 31.451 |  | 0.003 | 0.196 |
| Chl. b (mg/gfw) |  | 0.053 | 54.538 |  | 0.043 | 43.639 |  | 0.002 | 1.823 |
| Total Chl. (mg/gfw) |  | 0.259 | 19.307 |  | 1.076 | 80.054 |  | 0.009 | 0.639 |
| Carotenoids (mg/gfw) |  | 0.070 | 18.742 |  | 0.304 | 80.816 |  | 0.002 | 0.442 |
| Proline (mg/gfw) |  | 0.0015 | 73.356 |  | 0.0006 | 26.633 |  | 0.00000023 | 0.011 |
| Protein (mg/gfw) |  | 0.000048 | 8.506 |  | 0.000475 | 83.928 |  | 0.0000428 | 7.567 |
| Sugars (mg/gfw) |  | 0.121 | 67.838 |  | 0.054 | 30.601 |  | 0.003 | 1.561 |
| Phenol (mg/100gfw) |  | 181.732 | 72.228 |  | 69.003 | 27.425 |  | 0.873 | 0.347 |
| Essential oil (%) |  | 2.955 | 66.45 |  | 1.126 | 25.31 |  | 0.367 | 8.24 |

Based on the mean comparison results, different sickleweed populations were placed in different groups, which indicates the diversity and increased chance of selection for breeders. The results of the mean comparison for the studied traits showed that there is a big difference between different populations of sickleweed and this situation increases the chance of choosing favorable sickleweed populations for the breeder. The Ard-Shaban population had the highest values for essential oil, phenol, total chlorophyll, and Chl. a, and Chl. traits, and follow in the Qaz-Ilan population (Table 5). Therefore, these populations can be recommended for planting in different areas due to the high performance of photosynthesis, which plays a role in increasing production.

| **Table 5. The mean comparison of physiological and biochemical studied traits for sickleweed populations using Duncan's method.** | | | | | | | | | |
| --- | --- | --- | --- | --- | --- | --- | --- | --- | --- |
| Populations | Traits | | | | | | | | |
|  | Essential oil (%) | Phenol | Sugars (mg/gfw) | Protein (mg/gfw) | Proline (mg/gfw) | Carotenoids (mg/gfw) | Total Chl. (mg/gfw) | Chl. b (mg/gfw) | Chl. a (mg/gfw) |
| Kivananat | 8.90ab | 53.545h | 5.656c | 0.684bc | 0.261i | 4.152f | 9.597h | 2.216h | 7.381g |
| Bavaleh | 7.69cd | 53.411h | 5.712bc | 0.683bc | 0.271f | 4.597d | 10.892d | 2.325fg | 8.377d |
| Sahneh | 8.55a-c | 49.793i | 5.398d | 0.6706d | 0.287e | 4.778b | 8.173k | 2.074i | 7.228h |
| Chaharduli | 7.32de | 44.995j | 5.006f | 0.697a | 0.245k | 3.417j | 9.602h | 2.401e | 6.099m |
| Panjeh Ali | 5.43f | 66.269f | 5.016f | 0.638f | 0.227mn | 4.848a | 10.516e | 2.261gh | 8.255e |
| Bolbanabad | 4.36gh | 69.503e | 4.836g | 0.621g | 0.249j | 3.014l | 9.778g | 2.027i | 7.752f |
| Amirabad | 6.52e | 86.645b | 5.013f | 0.659e | 0.266h | 3.963g | 9.094j | 2.218h | 6.876j |
| Gerger-e Sofla | 7.11de | 81.538c | 5.272e | 0.676cd | 0.228m | 3.650i | 11.714b | 2.329f | 7.116i |
| Qaleh Gah | 5.26fg | 88.149a | 5.424d | 0.693ab | 0.269g | 4.167f | 9.182j | 2.492d | 6.224l |
| nemat abad auliya | 4.18h | 65.539f | 4.699h | 0.656e | 0.294d | 3.845h | 10.009f | 2.717c | 7.293h |
| Seylatan | 4.03h | 73.589d | 5.201e | 0.679cd | 0.227n | 3.231k | 9.588h | 2.516d | 6.791k |
| Ilan | 9.25a | 61.788g | 4.817g | 0.659e | 0.237l | 4.697c | 9.350i | 2.797b | 6.858kj |
| Shaban | 9.35a | 43.489k | 5.949a | 0.676cd | 0.366a | 4.907a | 9.441i | 2.958a | 9.384b |
| Khalajastan | 6.59e | 50.555i | 5.768b | 0.635f | 0.310c | 4.317e | 11.282c | 2.415e | 8.867c |
| Deylaman | 8.28bc | 66.685f | 5.033f | 0.642f | 0.342b | 4.141f | 12.482a | 2.919a | 9.562a |
| The treatments that have the same letters do not differ statistically at the 1% level. | | | | | | | | | |

The results of correltion analysis are presented in Fig 2. Based on the results, there was a significant positive correlation between essential oils and sugars, proline, carotenoids and chlorophylls. There was also a negative correlation between essential oils and phenols. There was a positive correlation between sugars and protein, proline and carotenoids and Chl. a.

**[Fig 2 near here]**

Principal component analysis was used before cluster analysis to clarify the relative importance of the variables that contribute to the cluster. The results of PCA for the studied traits are shown in Table 6. The eigenvalues obtained from components 1 to 3 justified 97.6, 1.2, and 0.85% of the total variance, respectively. The coefficients of the eigenvectors obtained from the first component showed that the phenol trait was the most important trait for clustering in this component. In the second component, Essential oil and Carotenoids traits, and in the third component, Total Chl. and Chl. a traits were the most important traits. The distribution of the studied populations based on the first two components is shown in Fig 3. The studied populations were divided into three groups. Populations Kur-Gerger-e, Sofla, Kur-Amirabad, and Kur-Qaleh Gah were in the first group, populations Kur-Panjeh Ali, Kur-Seylatan, Kur-Nemat abad auliya, Kur-Bolbanabad, Ham-Chaharduli, and Qom-Khalajastan were in the second group, and the rest of the populations were in the third group. Since the role of phenol in the first component and the role of essential oil and carotenoids in the second component was positive and effective. Therefore, the populations placed in the first area of the biplot are suitable populations for increasing the percentage of essential oil as well as carotenoids and phenol. In addition, biochemical and physiological traits play a role in stress tolerance, therefore the first area of the biplot of PC1 and PC3 is suitable. The Gilan-Deylaman and Kur-Gerger-e Sofla populations were placed in this area and identified as the superior populations of this study and can be used in breeding programs for tolerance.

| **Table 6. The results of four principal component analyses for traits** | | | | |
| --- | --- | --- | --- | --- |
| Traits | PC1 | PC2 | PC3 | PC4 |
| Essential oil | -0.06961 | 0.93826 | -0.25872 | -0.15457 |
| Phenol | 0.99697 | 0.07436 | -0.00513 | 0.019862 |
| Sugars | -0.01111 | 0.072748 | 0.022018 | 0.15669 |
| Protein | -0.00014 | 0.001947 | -0.00908 | -0.01002 |
| Proline | -0.00115 | 0.007903 | 0.010366 | 0.026353 |
| Carotenoids | -0.01557 | 0.19925 | -0.01841 | 0.46504 |
| Total Chl. | 0.005571 | 0.16237 | 0.75521 | -0.56126 |
| Chl. b | -0.0026 | 0.053882 | 0.056897 | 0.025628 |
| Chl. a | -0.02838 | 0.19951 | 0.5987 | 0.64686 |
| Eigenvalue | 210.545 | 2.59209 | 1.82469 | 0.474473 |
| % variance | 97.593 | 1.2015 | 0.84579 | 0.21993 |

**[Fig 3 near here]**

To determine the degree of diversity among the studied sickleweed populations was used the cluster analysis method. Different cluster analysis methods with different distance criteria were used to determine the degree of diversity between the studied populations, and the results showed that the UPGMA method with Euclidean distance had the highest value of the Cophentic correlation coefficient (0.79), and therefore, the cluster analysis was performed with this method.

The results of cluster analysis showed that there was the lowest genetic distance (2.08) between the populations of Ker-Bavaleh and Ker-Kivananat and the highest genetic distance (44.97) was between the populations of Ard-Shaban and Kur-Qaleh Gah. As seen in Fig 4, the different populations of sickleweed are divided into three main groups. The first group included the population of Kur-Gerger-e, Sofla, Kur-Qaleh, Gah, and Kur-Amirabad, the second group included the population of Kur-Panjeh Ali, Kur Seylatan, Kur-Nemat abad auliya, Kur-Bolbanabad, Gilan-Deylaman, and Qaz-Ilan, and In the third group were the populations of Ker-Kivananat, Ker-Bavaleh, Ker-Sahneh, Ard-Shaban, Ham-Chaharduli, and Qom-Khalajastan. The results showed that the populations were placed in separate categories due to different genetic bases or other environmental factors, and it can be justified that the morphological traits were able to determine this distinction. The results of cluster analysis show the differences between the populations of each group and the similarities and kinships of the populations within each group. The reason for the difference between the populations of the groups can be due to the difference in the genetic structure or the effect of other environmental factors on the traits.

**[Fig 4 near here]**

Population grouping by PCA was highly consistent with the grouping of cluster analysis. The results of this experiment showed that the number of studied traits is different in different regions. This diversity and difference in the amount of biochemical and physiological traits of sickleweed plants in different regions of Iran and other parts of the world can be caused by different weather factors (light, temperature, precipitation, relative humidity, etc.), geographical factors (geographical height, longitude, and latitude), soil conditions (soil texture, materials and nutrients) and genetic factors. Climatic conditions such as light, altitude, and average temperature have a great effect on the production of chemical compounds in horticultural and medicinal products [37]. The amount and type of active substances in medicinal plants are determined by the guidance of both environmental and genetic factors [38].

The investigation of phytochemical and physiological traits in this research showed that there was a significant difference between the studied traits of phenol content, percentage of the essence, biochemical, and physiological traits in different regions of Iran, in such a way that populations Gilan-Deylaman and Kur-Gerger-e Sofla have the highest values for these evaluated traits. Separate studies have shown in different medicinal plants [11, 12, 14] that biochemical and physiological trait are influenced by different collection areas, which is consistent with the results of this study. In the study of Crataegus oxyacantha plants, it was shown that the place of growth, height, and type of organ has a significant effect on the amount of phenol and flavonoids and it was found that at higher altitudes, more phenol and flavonoid compounds are produced in the plant [39-41]. Of course, the difference in the quantitative amounts of phytochemical compounds, including phenolic and flavonoid compounds, among the populations of different regions can be caused by genetic diversity or ecological conditions governing the habitats [41, 42]. The choosing and developing plants with high medicinal potential, high yield, or resilience to environmental stress requires an understanding of the variance in biochemical and physiological properties of plants across different geographic and climatic locations. Understanding the ecological and evolutionary processes that influence plant diversity and environment adaption is also crucial [17, 18].

**Conclusion**

The results of the mean comparison showed that populations Ard-Shaban and Qaz-Ilan in terms of the evaluated traits and especially the percentage of essential oil were at the upper of the studied samples and selected as suitable populations. In addition, populations Gilan-Deylaman and Kur-Gerger-e Sofla were also identified as superior populations in terms of studied traits by cluster analysis and PCA. Since the high level of proline and biochemical and physiological traits in plants can play a role in plants' tolerance to stresses, therefore, populations with high values of these traits can be used in stress tolerance breeding programs. Therefore, in this study, populations Gilan-Deylaman and Kur-Gerger-e Sofla can be suitable populations for this purpose. In addition, the essential oil of this plant is used in the treatment of diseases, and therefore populations Ard-Shaban and Qaz-Ilan, which showed a high percentage of essential oil, can be used in breeding programs to increase the performance of this trait. In conclusion, due to the influence of climatic conditions including temperature, rainfall, soil type, and altitude, the biochemical and physiological characteristics of medicinal plants might differ dramatically across different areas. The therapeutic potential of medicinal plants is significantly impacted by the diversity in their biochemical and physiological characteristics. As a result, it's critical to comprehend how diverse geographical areas affect medicinal plants and to build plans for their production and application in the pharmaceutical sector. Future research should look at the molecular mechanisms underlying how various geographic regions affect medicinal plants and devise plans to improve those plants' capacity to adapt to various environmental factors. The selection, breeding, and cultivation of medicinal plants with high therapeutic potential and productivity across the globe would benefit greatly from the information provided by studies across different regions.

**Acknowledgments**

We gratefully acknowledge the Iran National Science Foundation (INSF) and Graduate University of Advanced Technology (Kerman, Iran) to support this project.

**Declarations**

**Ethics approval and consent to participate**

Not applicable.

**Consent for publication**

Not applicable.

**Competing interests**

The authors declare that they have no conflict of interest.

**Funding**

This work was supported by the Iran National Science Foundation (INSF) under grant number [99026928].

**Availability of data and materials**

The data used to support the findings of this study are included in the article.

**Author Contributions**

M.R. designed the research. M.R. and M.K. experimented and analyzed data. M.R., M.K., and J.N. wrote the manuscript and revised the manuscript. MR provided comments during the writing and revised it. The authors read and approved the final manuscript.

**References**

1.	Mohammadi A, Nazari H, Imani S, Amrollahi H. Antifungal activities and chemical composition of some medicinal plants. J Mycol Med. 2014;24(2):1-8.

2.	Alwala S, Suman A, Arro JA, Veremis JC, Kimbeng CA. Target region amplification polymorphism (TRAP) for assessing genetic diversity in sugarcane germplasm collections. Crop Sci. 2006;46(1):448-55.

3.	Li Y, Kong D, Fu Y, Sussman MR, Wu H. The effect of developmental and environmental factors on secondary metabolites in medicinal plants. Plant Physiol Biochem. 2020;148:80-9.

4.	Bourgaud F, Gravot A, Milesi S, Gontier E. Production of plant secondary metabolites: a historical perspective. Plant Sci. 2001;161(5):839-51.

5.	Pardee GL, Inouye DW, Irwin RE. Direct and indirect effects of episodic frost on plant growth and reproduction in subalpine wildflowers. Glob Change Biol. 2018;24(2):848-57.

6.	Curry JP. Grassland invertebrates: ecology, influence on soil fertility and effects on plant growth. USA: Springer Science & Business Media; 1993.

7.	Korman BL. Biology and ecology of sickleweed (*Falcaria vulgaris*) in the fort pierre national grassland of south Dakota [Thesis]. USA: South Dakota State University; 2011.

8.	Bhandari H, Bhanu A, Srivastava K, Singh M, Shreya A, Hemantaranjan. Assessment of genetic diversity in crop plants - an overview. Adv Plants Agric Res. 2017;7(3):279-86.

9.	Rattan RS. Mechanism of action of insecticidal secondary metabolites of plant origin. Crop Prot. 2010;29(9):913-20.

10.	Cseke LJ, Kirakosyan A, Kaufman PB, Warber S, Duke JA, Brielmann HL. Natural products from plants. Florida, USA: CRC press; 2016. 569p p.

11.	Abdelmajeed NA, Danial EN, Ayad HS. The effect of environmental stress on qualitative and quantitative essential oil of aromatic and medicinal plants. Arch des Sci. 2013;66(4):100-20.

12.	Lianopoulou V, Bosabalidis AM, Patakas A, Lazari D, Panteris E. Effects of chilling stress on leaf morphology, anatomy, ultrastructure, gas exchange, and essential oils in the seasonally dimorphic plant Teucrium polium (Lamiaceae). Acta Physiol Plant. 2014;36(8):2271-81.

13.	Sharafzadeh S, Khosh-Khui M, Javidnia K. Effect of nutrients on essential oil components, pigments and total phenolic content of lemon balm (Melissa officinalis L.). Adv Environ Biol. 2011;5(4):639-46.

14.	Vojodi Mehrabani L, Kamran RV, Hassanpouraghdam MB, Pessarakli M. Zinc sulfate foliar application effects on some physiological characteristics and phenolic and essential oil contents of Lavandula stoechas L. under sodium chloride (NaCl) salinity conditions. Commun Soil Sci Plant Anal. 2017;48(16):1860-7.

15.	ZarghamiMoghaddam P, Mazandarani M, Zolfaghari M, Badeleh M, Ghaemi E. Antibacterial and antioxidant activities of root extract of Onosma dichroanthum Boiss. in north of Iran. Afr J Microbiol Res. 2012;6(8):1776-81.

16.	Arraouadi S, Badri M, Jaleel CA, Djébali N, Ilahi H, Huguet T, et al. Analysis of genetic variation in natural populations of Medicago truncatula of southern Tunisian ecological areas, using morphological traits and SSR markers. Tropical Plant Biology. 2009;2:122-32.

17.	Basit A, Saeed S, Ahmed A, Baloch N, Ali AZ. Genetic structuring of Ephedra an important medicinal plant from low to high altitudinal zones of Balochistan, Pakistan. Biodiversitas Journal of Biological Diversity. 2021;22(2):713-8.

18.	Gul J, Saeed S, Ahmed A, Basit A, Rehman A. Genetic diversity and morphological variation of Pinus gerardiana along the environmental gradient from Zhob, Balochistan, Pakistan. Nusantara Bioscience. 2021;13(1):121-8.

19.	Rehman A, Saeed S, Ahmed A. Genetic diversity and population structure of Seriphidium Sub-genus of Artemisia from different terrains of Balochistan, Pakistan. Biodiversitas Journal of Biological Diversity. 2021;22(5):2658-64.

20.	Yücedağ C, Müller M, Gailing O. Morphological and genetic variation in natural populations of Quercus vulcanica and Q. frainetto. Plant Systematics and Evolution. 2021;307:1-15.

21.	Fick SE, Hijmans RJ. WorldClim 2: new 1km spatial resolution climate surfaces for global land areas. International Journal of Climatology. 2017;37(12):4302-15.

22.	Sudhakar P, Latha P, Reddy P. Phenotyping crop plants for physiological and biochemical traits. USA: Academic Press; 2016.

23.	Arnon DI. Copper enzymes in isolated chloroplasts. Polyphenoloxidase in *Beta vulgaris*. Plant Physiol. 1949;24(1):1.

24.	Price A, Hendry G. Iron‐catalysed oxygen radical formation and its possible contribution to drought damage in nine native grasses and three cereals. Plant Cell Environ. 1991;14(5):477-84.

25.	Bates L, Waldren R, Teare I. Rapid determination of free proline for water-stress studies. Plant Soil. 1973;39(1):205-7.

26.	Bradford MM. A rapid and sensitive method for the quantitation of microgram quantities of protein utilizing the principle of protein-dye binding. Anal Biochem. 1976;72(1-2):248-54.

27.	Somogyi M. Notes on sugar determination. J Biol Chem. 1952;195:19-23.

28.	Malik CP, Singh M. Plant enzymology and histo-enzymology. New Delhi, India: Kalyani Publishers; 1980. 434 p.

29.	European Pharmacopoeia. European Directorate for the Quality of Medicines & HealthCare, Guide for the elaboration of monographs on herbal drugs and herbal drug preparations. France: Council of Europe; 2007.

30.	SAS-Institute-Inc. Base SAS 9.4 Procedures Guide: Statistical Procedures, Third Edition. USA: SAS Institute Inc., Cary, NC; 2014.

31.	Hammer Ø, Harper D, Ryan P. PAST: Paleontological statistics software package for education and data analysis. Palaeontol Electron. 2001;4(1):e4, 9pp.

32.	Kassambara A, Mundt F. Factoextra: Extract and Visualize the Results of Multivariate Data Analyses. R Package Version 1.0.7. <https://CRAN.R-project.org/package=factoextra2020>.

33.	Rockwood LL. Introduction to population ecology. Chichester, West Sussex, England: John Wiley & Sons; 2015. 378 p p.

34.	Anacker BL, Strauss SY. The geography and ecology of plant speciation: range overlap and niche divergence in sister species. Proc Royal Soc B Biol Sci. 2014;281(1778):e20132980.

35.	Givnish TJ. Ecology of plant speciation. Taxon. 2010;59(5):1326-66.

36.	Pontarotti P. Evolutionary biology: Convergent evolution, evolution of complex traits, concepts and methods. Switzerland: Springer; 2016.

37.	Klein B, Perry A. Ascorbic acid and vitamin A activity in selected vegetables from different geographical areas of the United States. J Food Sci. 1982;47(3):941-5.

38.	Urbonavičiūtė A, Jakštas V, Kornyšova O, Janulis V, Maruška A. Capillary electrophoretic analysis of flavonoids in single-styled hawthorn (*Crataegus monogyna* Jacq.) ethanolic extracts. J Chromatogr A. 2006;1112(1-2):339-44.

39.	Farokhi H, Asgharzadeh A, Kazemi Samadi M. Investigation of some growth and physiological traits changes and heavy metals accumulation in saffron (Crocus sativus L.) under different climates cultivation. Eco-phytoch J Med Plants. 2022;10(1):114-29.

40.	Guedri Mkaddem M, Zrig A, Ben Abdallah M, Romdhane M, Okla MK, Al-Hashimi A, et al. Variation of the Chemical Composition of Essential Oils and Total Phenols Content in Natural Populations of *Marrubium vulgare* L. Plants. 2022;11(5):612.

41.	Saberi M, Kamali N, Tarnian F, Sadeghipour A. Investigation Phenol, Flavonoids and Antioxidant Activity Content of Capparis spinosa in Three Natural Habitats of Sistan and Baluchestan Province, Iran. J Rangel Sci. 2022;12(2):191-204.

42.	Singh SM, Maurya VK. Phytochemical investigations and medicinal uses of *Achyranthes aspera* L.: A medicinal plant. In: Pandey RR, Kumar J, editors. Curr Trends Med Chem. India: Thanuj International Publishers; 2022. p. 139-54.

**Figure Captions**

**Fig 1. The region cluster analysis of Sickleweed population by bioclimatic parameters**

The name of population is given in Table 1.

**Fig 2. The correlation diagram of studied traits in sickleweed populations**

**Fig 3. The biplot grouping of studied population based on four principal component analysis**

**Fig 4. The cluster analysis dendrogram of studied population based on UPGMA method**
