# Supplementary material for: Evaluation of biochemical, physiological traits and percentage of essential oil of sickleweed (Falcaria vulgaris) population in different geographical and climatic regions
Source: PLoS One. 2023 Jun 21;18(6):e0287480. doi: 10.1371/journal.pone.0287480 (PMC10284402; doi:10.1371/journal.pone.0287480)
Supplement: Attachment — Submitted filename: Response to Reviewers.docx [file pone.0287480.s004.docx]

**Dear Editor**

Thank you for your useful comments and suggestions on the language and structure of our manuscript. We have modified the manuscript accordingly and showed in the revised MS by yellow highlighted and Track Changes. Also, detailed corrections are listed below point by point.

Thanks once again.

Best Regards

On behalf of the authors

**Reviewer comments**

**Reviewer #3:**

Authors have done nice work on the 15 different sickleweed populations from seven provinces of the Iran. The data obtained will be helpful to choose sickleweed population in breeding programs. The work showed that there is variation among populations which is very important for further planning of research on sickleweed.

I recommend the paper for publication after following corrections.

Do following corrections:

Line Nos.:139, hour to h

Answer: Corrected in text

142, minute to min

Answer: Corrected in text

146-147, degree Celsius to OC

Answer: Corrected in text

154, two milliliters to 2 ml

Answer: Corrected in text

156, degrees Celsius to OC

Answer: Corrected in text

157, minute to min

Answer: Corrected in text

157, two milliliters to 2 ml

Answer: Corrected in text

199, started line with 19 bioclimatic indicators instead start with, “Bioclimatic indicators (19)”.

Answer: Corrected in text

Conclusion is very lengthy and bit vague. Make is concise and to the point.

Answer: The conclusion was summarized.

**Reviewer #4**

The authors should address the following:

What are the ages of different Sickleweed plant obtained from each region?

Answer: Corrected and revised in text. Although Sickleweed has been described as annual, biennial or perennial [13], but according to the authors' observations and evaluations, it was annual.

What is the reference of formula 1, 2, 3 and 4?

Answer: Corrected in text and the reference added to text.

Give some brief information for Elbow technique which used in clustering the regions?

Answer: Added in text and describe this method.

Conclusion should be shortened

Answer: The conclusion was summarized.

See the attached MS file and consider all required corrections

Answer: All required corrections in text based on suggestion and Also, suggested references were added to the text.

We would like to give you a special thanks for your careful reading of the whole MS and also for your meaningful corrections that helped us a lot to improve our manuscript significantly. We have revised the manuscript according to your suggestions and the other reviewer’s suggestions and highlighted.

Best Regards

On behalf of the authors
